# Decline in influenza cases in Mexico after the implementation of public health measures for COVID-19

**DOI:** 10.1038/s41598-021-90329-w

**Published:** 2021-05-24

**Authors:** Daniel Arellanos-Soto, Gerardo Padilla-Rivas, Javier Ramos-Jimenez, Kame Galan-Huerta, Sonia Lozano-Sepulveda, Natalia Martinez-Acuña, Consuelo Treviño-Garza, Roberto Montes-de-Oca-Luna, Manuel de-la-O-Cavazos, Ana Maria Rivas-Estilla

**Affiliations:** 1 Department of Biochemistry and Molecular Medicine, School of Medicine and Hospital Universitario “Dr. Jose E. Gonzalez”, Autonomous University of Nuevo Leon, Ave. Francisco I. Madero y Ave., Gonzalitos S/N, Col. Mitras Centro, 64460 Monterrey, Nuevo Leon Mexico; 2 Department of Internal Medicine, Infectious Disease Service, School of Medicine and Hospital Universitario “Dr. Jose E. Gonzalez”, Autonomous University of Nuevo Leon, Monterrey, Nuevo Leon Mexico; 3 Department of Pediatrics, School of Medicine and Hospital Universitario “Dr. Jose E. Gonzalez”, Autonomous University of Nuevo Leon, Monterrey, Nuevo Leon Mexico; 4 Department of Histology, School of Medicine and Hospital Universitario “Dr. Jose E. Gonzalez”, Autonomous University of Nuevo Leon, Monterrey, Nuevo Leon Mexico; 5 Secretariat of Health of Nuevo Leon State, Monterrey, Nuevo Leon Mexico

**Keywords:** Epidemiology, Disease prevention, Infectious diseases

## Abstract

Mexico took swift action and has strictly followed mitigation measures to prevent the spread of coronavirus disease, COVID-19. In this study we compared influenza activity indicators in our country after the implementation of public health measures for COVID-19. We compared indicators of influenza activity in 2020 before and after public health measures were taken to reduce COVID-19 with the corresponding indicators from three preceding years and the immediate one, and the potential decrease in seasonal influenza cases/deaths. Nationwide surveillance data revealed a drastic decline in influenza diagnosis in outpatient clinics and public hospitals, influenza positivity rates of clinical specimens, and confirmed severe cases during the following 10 weeks of 2020 as lockdown activities and control measures were established compared with the same period of 2019. Our results suggest that the measures taken for COVID-19 were effective in reducing the spread of other viral respiratory diseases as influenza in our country.

## Introduction

Public health measures, including public education and physical distancing, were implemented in Mexico to reduce transmission of COVID-19 after the first few cases were reported in epidemiological week 3 (January 13, 2020) and public awareness was increased^1^. We examined the effect of these COVID-19 measures on influenza incidence as a proxy for determining the overall potential reduction in respiratory virus transmission.

Mexico, like other Latin American countries, established population confinement and a reduction in its economic activity to face the coronavirus pandemic and the respiratory disease it generates, COVID-19. The Mexican Ministry of Health established a package of prevention measures called "National Protocol for Healthy Distance", effective as of Monday, March 23, 2020, to prevent the spread of COVID-19 in the community^2^. These control strategies focused on social distancing and basic prevention measures and were aligned with those used for other common respiratory viral infections such as influenza: use of face masks, avoiding contact when greeting, hand washing, cough etiquette, temperature monitoring, home confinement, and avoiding close contact with sick individuals. In addition to this, cancellation of large-scale events, the lockdown of activities at all levels of education and workplaces (e.g., segregated teams and home-office working wherever possible) and non-essential activities of the public, social, and private sectors were temporarily suspended, and the reprogramming of massive events was raised. Also, intensive public health education on public and personal hygiene and social responsibility was established^3^.

We compared influenza activity indicators in 2020 before and after public health measures were taken to reduce COVID-19 with the corresponding indicators from the three preceding years and the immediately previous one, as well as the involvement in the decrease of seasonal influenza cases.

## Methods

We obtained routine sentinel surveillance data on influenza-like illnesses (ILI) from the official reports of the Mexican Ministry of Health and the National Centre of epidemiological surveillance for all weeks since 2016^4^. ILI were defined as fever (> 38 °C) and cough. ILI samples were tested by using influenza-specific real time RT-PCR assay to identify influenza virus infection and then percentage of influenza cases were registered per week. Deaths caused by confirmed influenza virus infection analyzed were taken from the total confirmed cases reported in the official weekly epidemiological reports, which are open and available to the public.

We evaluated whether the influenza trends in the 2019–2020 season were different before and after the COVID-19 National Protocol for Healthy Distance was implemented compared to previous seasons by using a quasi-experimental difference-in-difference design. We extracted weekly reports of seasonal influenza cases data from the 2016–2017 season to the 2019–2020 season from epidemiological (EPI) week 40 to EPI week 30, using the open-access databases of the public health authority^4^.

We compared the number of influenza cases in season 2019–2020 and the average of the corresponding periods in the previous three influenza seasons (2016–2019). The normal distribution of the data was analyzed using a Kolmogorov–Smirnov test, finding that the data presented a normal distribution and the LINE condition was met, allowing the use of the t test to perform the statistical analysis for each EPI week using IBM SPSS Statistics for Windows, Version 23.0^5^.

### Ethics approval

Not necessary, the data analyzed is publically available on Secretariat of Health of México databases.

### Consent to participate

Not necessary. We analyzed data publically available on Secretariat of Health of México databases.

### Consent for publication

All the authors give their consent for this work publication. This work has not been published previously), and it is not under consideration for publication elsewhere.

## Results

In Mexico, influenza incidence follows a yearly bimodal pattern: the influenza season and the inter-seasonal period. The influenza season spans from October to March, including autumn and winter (2 years, weeks 40–20).

**Figure 1:**
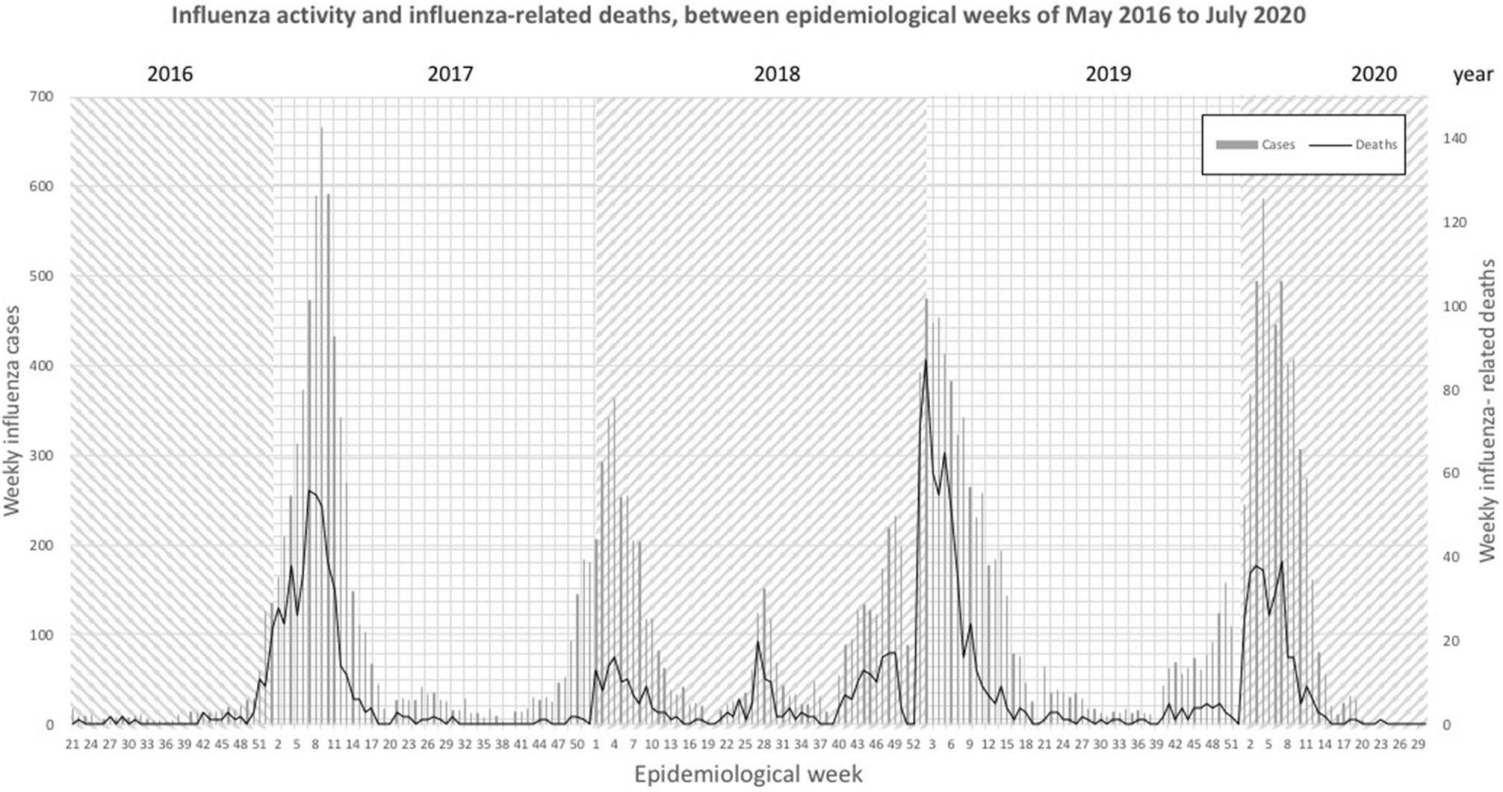
Trends of influenza activity and influenza-related deaths during the 2019–2020 season compared with average of the preceding 3 years (between EPI weeks of May 2016 to July 2020).

We compared indicators of influenza transmissibility (confirmed cases and deaths) in 2020 against the average from corresponding periods in the three preceding influenza seasons (2016–2020). Figure 1 shows trends in seasonal influenza cases from the 2016–2017 season to the 2019–2020 season in Mexico. The average influenza activity peaked in EPI weeks 3–7 in the last four influenza seasons and declined by week 15 every year (Fig. 1). Annually in our country, on average, around 500–1000 people die from laboratory-confirmed influenza (Fig. 1)^6^.

**Figure 2:**
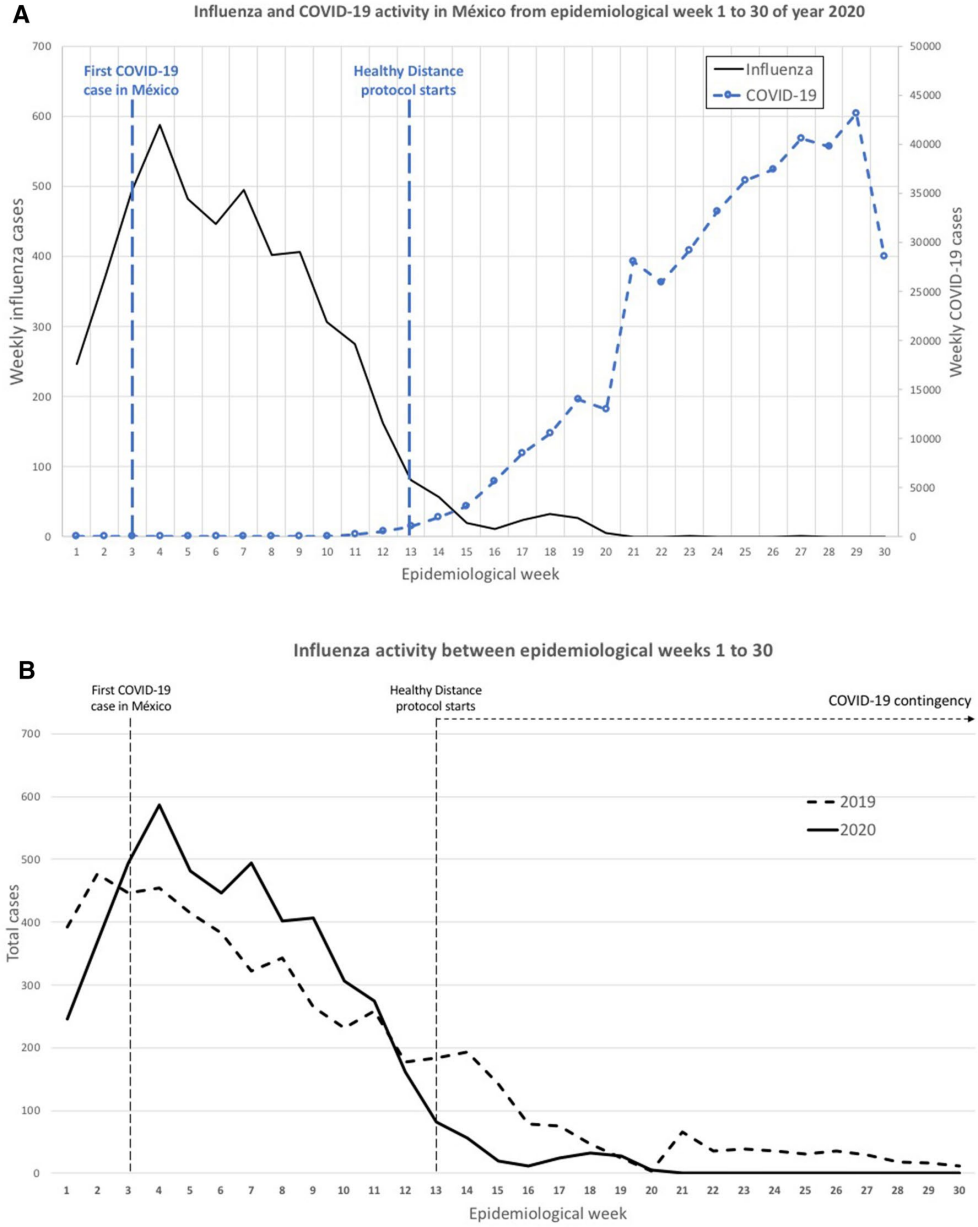

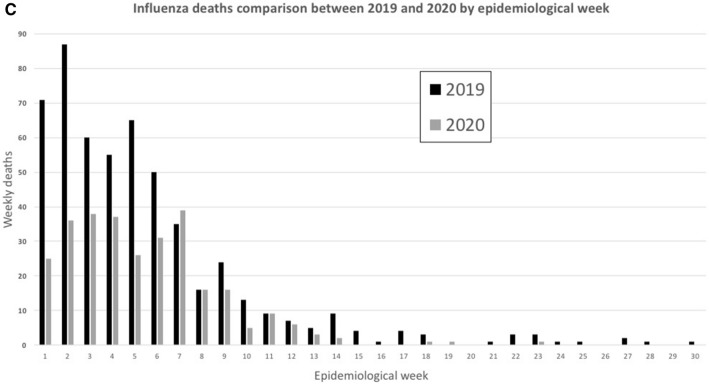
Trends of influenza activity during the 2019–20 season. (**A**) Influenza and COVID-19 activity in México from EPI weeks 1–30 of year 2020; (**B**) Influenza activity between EPI weeks 1–30; (**C**) Influenza deaths comparison between 2019 and 2020 by EPI week.

In Mexico, the number of seasonal influenza cases in the 2019–2020 season was lower after COVID-19 transmission compared to previous years (Figs. 1, 2A). We observed the peak of influenza cases in week 4. Particularly, the average of influenza cases at week 4 was significantly different (p < 0.05) from the previous two influenza seasons (587 vs 357.33 ± 99.6; respectively).

The number of influenza cases decreased below the average of previous years in EPI week 12 (2017–2019). In addition, we observed that after EPI week 12, in Mexico, 9 weeks had passed since the detection of the first SARS-CoV-2 case and the number of COVID-19 cases was increasing (Fig. 2A). The healthy distance protocol was officially established in EPI week 13. It was observed that simultaneously the cases of influenza were decreasing and were unusually low after week 20 compared to previous years. Even when measures to contain the transmission of the new respiratory disease were established (EPI week 13), the number of COVID-19 cases increased while prevalence of influenza declined (Fig. 2A). In fact, the number of influenza cases in 2020 decreased significantly (p < 0.05) from EPI week 22 to 30 compared to the seasons 2016–2019 (Fig. 2B). It is important to highlight that this phenomenon indicates a significant difference in the transmission mechanisms and the pathogenesis of these two diseases.

We performed a weekly paired difference *t* test using IBM SPSS Statistics for Windows, (Version 23.0)^5^. We found that the percentage of influenza cases decreased by 64% (p = 0.001) and the estimated daily number of influenza cases decreased by 76% (p = 0.002) in EPI week 20 of season 2019–2020 compared with the preceding years (Fig. 2B). Additionally, before EPI week 20, the number of deaths in 2020 from influenza per EPI week was statistically similar (p > 0.05) to the average of the seasons 2016–2019 (Figs. 1, 2C). In contrast, we saw no significant changes in deaths, only in the percentage of influenza positivity (31%; p = 0.008), in EPI weeks 21–30 of 2019–2020 compared with preceding seasons (Figs. 1, 2C).

The COVID-19 epidemic has altered social and health behaviors, resulting in an unexpected reduction of seasonal influenza cases. Influenza disease has not been detected by our national laboratories since May 8th. This dramatic decrease is unlikely to be related to climatic variations as the number of laboratory‐confirmed influenza A were similar during the same weeks in 2019 (relative risk 1.3, 95% CI 0.4–3.8, p = 0.7), which was consistent with the typical transmission pattern in Mexico.

**Figure 3:**
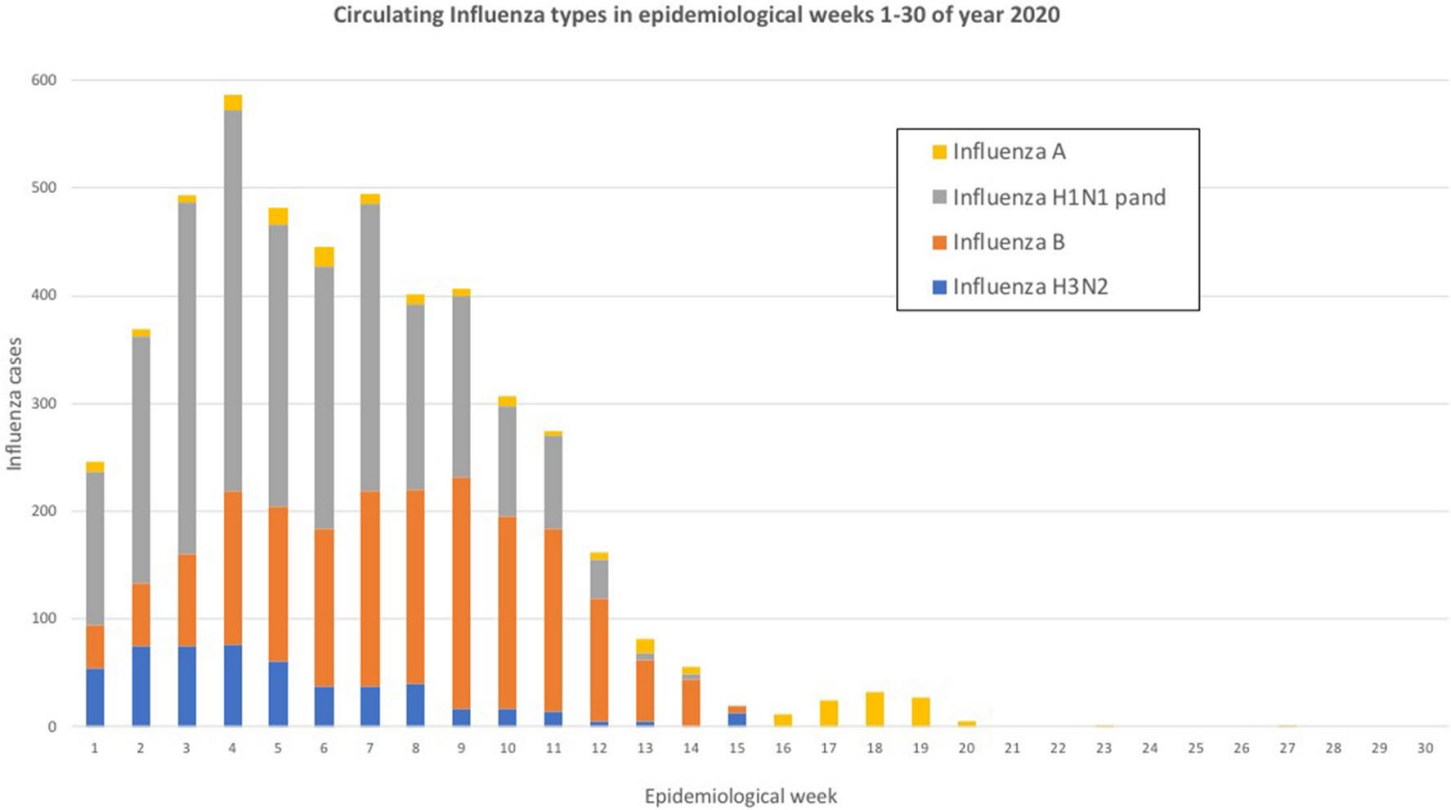
Circulating Influenza types in EPI weeks 1–30 of year 2020.

In addition, both the number of influenza strains isolated from clinical specimens in commissioned laboratories and the positivity rate dropped drastically in 2020; the trends were different from 2019 (p < 0.05 for both). In the 2019–2020 influenza season (weeks 40–30), circulation of influenza A (H1N1) pdm09 (47%), B (30%), A (H3N2) (19%) and influenza A non-subtypeable (4%) was observed. In week 20, the cumulative positivity rate was 8.9%, which decreased by week 30 with only two cases of non-subtypeable Influenza A identified (Fig. 3).

## Discussion

According to the WHO, the lethality of COVID-19 is higher than for seasonal influenza (usually well below 0.1%). COVID-19 mortality seems to differ according to the region^6^. Until now, it is estimated that the crude mortality ratio (the number of reported deaths divided by the reported cases) is between 3–4%; therefore, the infection mortality rate (the number of reported deaths divided by the number of infections) will be lower. However, for both diseases, mortality is determined to a large extent by regional access to and quality of healthcare services.

In our country, the functional healthcare and surveillance systems installed, the government’s efforts to identify ILI cases during the COVID-19 pandemic, and adequate laboratory capacity guarantee an appropriate influenza testing and reporting of results. As reported by other countries, public health initiatives to control COVID-19 spread probably helped to decrease influenza transmission and number of cases in May 2020 because both viruses share the same transmission mechanisms through the respiratory route and contact, although with different efficiency giving a basic reproduction number (R_0_) for COVID-19 higher than that of seasonal influenza, then a reduction in transmission could reduce the impact of COVID-19, thereby preventing the increase of mortality. The modeling of the effective reproduction number for COVID-19 in Mexico in May 2020 (National Secretariat of Health) at 0.5–1, is lower (55–77% less) than the mean estimated R_0_ for this virus (2.2)^7^. The observed 61% reduction in influenza transmission is consistent with the information above^8^.

Due to the measures implemented in our country to contain the COVID-19 cases, there were severe changes in the availability of medical care and assistance, including hospitalization. Avoidance of medical care during this period may be a major confounder in interpreting our results. It is important to note that due to the similarity in symptoms between COVID-19 and influenza and the low number of COVID-19 patients in Mexico (< 200 cases as of March 21, 2020), ILI patients would seek help for a differential diagnosis. The reduction of the availability of medical care also did not explain the lower number of severe influenza cases seen in 2020 (Fig. 2C). Therefore, we believe that the decline in influenza activity in Mexico in 2020 is the result of the strict control measures that were put in place in response to COVID-19.

During the last winter season, European countries and the North American region (United States, Canada and Northern Mexico) have reported fluctuations in the number of cases of seasonal influenza different from experienced in previous seasons (since EPI week 4, 2019–2020 season) European countries and North America region (United States, Canada and North Mexico) have reported similar trends in the fluctuation of seasonal influenza cases from those experienced in previous seasons. In contrast, some Asian regions experienced different trends in influenza incidence during this season, from those experienced in past years^9,10^. The total number of patients infected with influenza in countries like Hong Kong, Japan, and Taiwan was significantly lower after EPI week 4 of the 2019–2020 season than in previous influenza seasons^11^.

We can mention some limitations in our study. First, due to the seasonal bimodal pattern of influenza incidence in Mexico, it is expected that influenza cases diminish around March–May. However, this year the number of cases fell to almost zero. Second, it is important to consider an underdiagnosis of ILI’s due to the fear of leaving home in search of medical attention and catching coronavirus, as well as being classified as COVID-19, all this contributes to generating an altered pattern^11^. Third, if we consider a similar dynamic for COVID-19, we will also have to record a higher number of cases for COVID-19 due to the underdiagnosis and the presentation of asymptomatic cases^12,13^.

In conclusion, we found a marked decline in influenza cases in Mexico after the implementation of public health measures for COVID-19. Definitively, the containment measures to reduce the risk of contagion for the new respiratory disease was additionally efficient for influenza, but with greater magnitude. This is partly explained by the different transmission mechanisms of these two respiratory diseases, the virulence of each viral agent, the absence of immunity in the population, and the diverse virological factors inherent in each pathogen. Our results suggest that such measures are useful in reducing the spread of viral respiratory diseases and their establishment mitigate the impact of the COVID-19 pandemic, especially now that we are entering into a new influenza season.

## Data Availability

Data analyzed in this work is publically available.
